# Social Needs and Acculturation as Predictors of Emotional Problems and Perceived Stress Among Latinx Mothers with Low Income

**DOI:** 10.1007/s10903-022-01430-9

**Published:** 2022-11-24

**Authors:** Victoria F. Keeton, Janice F. Bell, Laura M. Gottlieb, Christiana Drake, Matthew Pantell, Danielle Hessler, Holly Wing, Erik O. Fernandez y Garcia

**Affiliations:** 1 Department of Obstetrics, Gynecology, and Reproductive Sciences, University of California, San Francisco, 490 Illinois St, Box 2930, 94143 San Francisco, CA USA; 2 University of California, Davis, Betty Irene Moore School of Nursing, Sacramento, USA; 3 Department of Family and Community Medicine, University of California, San Francisco, USA; 4 Department of Statistics, University of California, Davis, USA; 5 Department of Pediatrics, University of California, San Francisco, USA; 6 Center for Health and Community, University of California, San Francisco, USA; 7 Department of Pediatrics, University of California, Davis, USA

**Keywords:** Social needs, Stress, Acculturation, Mothers, Latinx

## Abstract

Associations between household social needs, acculturation, and emotional health remain understudied, particularly among Latinx mothers. We analyzed baseline survey data from 455 Latinx mothers in a previous study. Using multinomial regression, we examined whether emotional problems and perceived stress were associated with household social needs and acculturation. Almost half the sample reported four or more household social needs. Social needs cumulatively and independently predicted increased odds of frequent emotional problems or perceived stress. Lower acculturation predicted lower odds of frequent emotional problems. There is increased risk for emotional problems and stress in low-income Latinx mothers who experience high social need. Integrated social service and mental health care models may be one way to improve health outcomes. More research is needed to understand how clinical settings can leverage unique cultural protective factors to address the social and emotional health needs of Latinx mothers.

## Introduction

### Background

Latinx women in the U.S. experience high rates of emotional disorders [1–5]. These conditions contribute to other psychiatric disorders, chronic physical health conditions, and poor quality of life [6, 7]. It is important to consider mothers specifically because of the intergenerational implications of poor emotional health, which is associated with poor health in their children [8–11] and harmful consequences into later life [12].

Higher levels of acculturation in immigrant or U.S.-born Latinx populations have been associated with worse mental health outcomes [1, 3, 5, 13]. The “Hispanic health paradox” posits that cultural factors rooted in the country of origin buffer the impact of U.S. societal factors that negatively influence health [5, 14–17]. For example, “familismo” emphasizes the importance of family loyalty and bonding in many Latinx cultures, and may help to diminish the impacts of physical or mental health stressors [3, 17–19]. With acculturation, these cultural factors and their protective effects may be lost. Concurrently, researchers have documented an increase in the negative health impacts stemming from discrimination experiences as immigrants integrate into their new environments [5, 17, 19–21].

Income poverty has been shown to be an important predictor of mental health outcomes in the U.S., generally, and for Latinx women, specifically [3, 22–24]. More recent literature has underscored that material hardship—disadvantage related to social needs such as food or housing [25]—is closely tied to health outcomes [26–30]. Studies have linked select social needs to negative emotional health outcomes in parents with low income [2, 27–30], though these are primarily large population studies that lack the ability to explore variation in ethnic subgroups, including the potential influence of cultural concepts of distress [31] or social desirability bias [32].

### Aims and Hypotheses

In this analysis, our primary aim was to explore associations between count of household social needs, acculturation, and self-reported frequency of emotional problems and perceived stress in a population of Latinx mothers with low income. Our hypothesis was that reports of more household social needs would predict greater frequency of emotional problems and perceived stress in mothers, especially among more acculturated subjects, even after controlling for sociodemographic characteristics. Our secondary aim was to explore whether specific social needs were associated with maternal frequency of emotional problems or perceived stress.

## Methods

### Study Design, Setting, and Sample

We performed a secondary analysis of cross-sectional baseline survey data obtained at enrollment in a randomized clinical trial (RCT) involving over 600 children and their caregivers who sought care in the pediatric urgent care health center of a public hospital in San Francisco, California. The RCT took place between July 2016 and June 2018; details of the RCT’s recruitment process and eligibility criteria are published elsewhere [33]. We limited the sample in this analysis to caregivers who identified as mothers (n = 549) and of “Hispanic or Latino origin or descent” (n = 455). The Committee for the Protection of Human Subjects at the University of California, Davis approved the study as exempt from full review because we used only de-identified data in these analyses.

### Measures and Variables

The mother was the unit of analysis for this study. At the time of enrollment in the RCT, mothers completed surveys (in English or Spanish) about themselves and their children, including questions about their demographic characteristics, social situation and needs, and their physical and mental health.

#### Outcome Variables

Two primary outcomes were examined: (1) frequency of “bother by emotional problems”; and (2) perception of stress.

The Patient-Reported Outcomes Measurement Information System 10-item Global Health scale (PROMIS Global®) [34] is reliable and valid for the assessment of physical and mental health in diverse [35, 36] and Spanish-speaking [35] populations. Mothers’ reports of emotional problems were measured using a single item from the PROMIS Global Mental Health sub-scale: “In the past 7 days, how often have you been bothered by emotional problems such as feeling anxious, depressed or irritable?” Answers are provided using a 5-point Likert scale from 0 (never) to 4 (always). The ends of the scale are similar conceptually, i.e. 0 (never), 1 (rarely), and 4 (often), 5 (always). As a reflection of that, each of these items had fewer responses than the middle choice, 3 (sometimes). We recoded the variable into three groups based on the face validity of the scale response options (never/rarely = reference, sometimes, often/always), to reflect the conceptual similarities and distribution of responses, which still enabled analysis of the spectrum between infrequent to frequent symptoms.

Maternal perceived stress was measured with the PSS-4, a shortened version of the Perceived Stress Scale [37], which measures adults’ perceived stress in their ability to control or handle life events occurring in the past month [38]. Items are scored on a five-point Likert scale ranging from 0 (never) to 4 (very often), where higher scores reflect higher perceived stress [38]. The PSS-4 tool is reliable and valid in English-speaking [38] and Spanish-speaking [39] participants. Because there are no formal cutoffs for scores on the 20-point PSS-4 summative scale, responses were divided into tertiles so that each contained a third of the sample responses, and each category was defined by the scores contained in each tertile (0–4 = reference, 5–8, 9 and higher).

#### Independent Variables

We tested associations with two primary predictor variables: (1) household social needs, and (2) mother’s level of acculturation.

Household social needs variables were constructed in two ways: (1) categorical by count of household needs (primary aim), and (2) select independent household needs (secondary aim). Category of household need was based on the total count of mothers’ “yes” responses to current concerns about 18 possible social issues for either themselves or members of their household. Type of needs included: *financial* (problems paying utility or medical bills, denied other income support, no health insurance), *housing* (difficulty finding housing, or habitability concerns), *food* (running out of food before having money to buy more), *transportation* (difficulty affording transportation or disability paratransit), *employment* (difficulty finding or problems with a job, disability interfering with work, difficulty obtaining unemployment benefits), *legal* (deportation, child support or family law issues), or *other* (no primary care provider, difficulty finding childcare or after-school activities, bullying or household mental health concerns). The total number of needs was then divided into three groups, the first with participants who reported no needs (none = ref), and the remaining participants divided into two relatively even groups (1–3 needs, 4 or more needs).

To minimize the risk of chance findings due to multiple comparisons, from the list of 18 needs we selected 7 of the most reported needs (those with a frequency of 30% or greater) as independent predictor variables for our secondary aim: problems paying utility bills, unstable housing, habitability problems (e.g., mold or infestations), running out of food, difficulty finding a job, difficulty affording transportation, or other legal issues (no = ref, yes).

Level of acculturation was measured by the mothers’ preferred language on the survey (English = reference, Spanish), where Spanish-preferring participants were considered to have a lower level of acculturation. Language has been considered a valid proxy measure of acculturation in research, particularly in secondary analyses when more multidimensional scales of acculturation were not included as part of the original study [3, 21].

#### Covariates

Covariates included sociodemographic and health characteristics known for associations with maternal mental health [2, 40], stress [22, 41, 42], or social needs [2, 8, 26, 43]. The following covariates were used in all models: mother’s age in years (18–24 = reference, 25–34, 35–44, 45 and older), child’s age in years (0–4 years, 5–17 years = reference), mother’s highest level of education (less than high school = reference, high school/general educational development [GED], technical school/some college, college graduate/graduate school), and mother’s employment status (employed = reference, unemployed). Finally, we included mother’s rating of her own physical health, via responses ranging on a 5-point scale from 0 (poor) to 4 (excellent) to one item from the Global Physical Health sub-scale of the PROMIS Global® instrument [44]: “In general, how would you rate your physical health?” (recoded into two groups as good/very good/excellent = reference, poor/fair).

It should be noted that in the original study only the child presenting for a visit was enrolled as a participant, and therefore the child’s age covariate refers only to the enrolled child. The total number of children under 18 years of age in the household was available in the original dataset, although no other descriptive data about the other children was collected to support the inclusion of this variable as a covariate. In post-hoc analyses we explored whether the number of children had any impact on our results but found no significant effects (results not shown). We also decided not to include income as a covariate because almost 90% of the full sample reported an annual household income under $50,000, and income was closely correlated with mother’s education. In the county of San Francisco in 2018, the low-income limit as defined by the U.S. Department of Housing and Urban Development was $82,200 for a single individual [45]. We therefore use this cut-off in describing the overall sample as having low income.

### Data Analysis

Data were analyzed using Stata, version 16 statistical software (College Station, TX, USA). Descriptive statistics summarized the sociodemographic and health characteristics of the sample and the dependent and independent variables, and chi-square tests compared characteristics of English vs. Spanish-preferring participants. In the analyses of our primary outcomes, although the outcome variables were ordinal they did not meet the proportional odds criterion. A multinomial regression model with less restrictive assumptions provided an adequate fit. We converted to categorical variables and used multinomial logistic regression to model frequency of maternal emotional problems (never/rarely = reference) or perceived stress score (0 to 4 = reference) as functions of household social needs and mother’s language, controlling for the sociodemographic and health covariates. Results from the multinomial models are odds ratios (ORs) comparing the emotional problems or perceived stress categories to the reference group. For our primary aim, each outcome was modeled with the categorical count of social needs. For our secondary aim, each outcome was modeled using one social need at a time along with language and the covariates, resulting in seven separate models.

## Results

### Demographic and Descriptive Characteristics

Sociodemographic and health characteristics are summarized for the sample (Table 1). Most participants preferred Spanish (85%). Overall, 22% reported being often or always bothered by emotional problems in the past week, with English-preferring participants significantly more likely than Spanish-preferring (32% vs. 20%, p < 0.01). The mean PSS-4 score was 5.53 (SD: 3.20, Range: 0–15; results not shown in table) and 31% of the sample had a high perceived stress score, with no significant difference between groups. The median count of social needs was 3 (Range: 0–18; Mean: 4.13, SD: 3.18; results not shown in table). Most of the sample reported having at least one social need (90%), and almost half reported having four or more needs (49%). The most reported needs were difficulty paying utility bills (39%), unstable housing (39%) and food insecurity (36%). Other legal issues, reported by 30% of participants, was the only independent need significantly different between groups, with Spanish-preferring more likely than English-preferring participants to report this need (31% vs. 18%, p = 0.03). Over half of the sample reported less than a high school education (58%) and of these, significantly more were Spanish-preferring (64% vs. 18%, p < 0.01). Most mothers rated their own physical health as good, very good, or excellent (63%).

**Table 1:** Sociodemographic and Health Characteristics of Latinx Mothers with Low-Income (N=455)

	All (n=455)	English (n=64)	Spanish (n=391)	*p*
**Bothered by Emotional Problems, n (%)**				
Never/Rarely	166 (36.48)	12 (18.75)	154 (39.39)	<0.01
Sometimes	188 (41.32)	31 (48.44)	157 (40.15)	
Often/Always	101 (22.20)	21 (32.81)	80 (20.46)	
**PSS-4 Score, n (%)**				
0 to 4	172 (37.80)	17 (26.56)	155 (39.64)	0.12
5 to 8	139 (30.55)	24 (37.50)	115 (29.41)	
9 to 20	144 (31.65)	23 (35.94)	121 (30.95)	
**Household Count of Social Needs, n (%)**				
None	42 (9.23)	5 (7.81)	37 (9.46)	0.79
1-3 needs	190 (41.76)	29 (45.31)	161 (41.18)	
4 or more needs	223 (49.01)	30 (46.88)	193 (49.36)	
**Most Frequently Reported Social Needs, n (%)**				
Problems paying bills	180 (39.56)	29 (45.31)	151 (38.62)	0.31
Unstable housing	178 (39.12)	31 (48.44)	147 (37.60)	0.09
Habitability problems (e.g., mold)	136 (29.89)	21 (32.81)	115 (29.41)	0.58
Running out of food	167 (36.70)	30 (46.88)	137 (35.04)	0.06
Difficulty finding a job	142 (31.21)	25 (39.06)	117 (29.92)	0.14
Difficulty affording transportation	143 (31.43)	23 (35.94)	120 (30.69)	0.40
Other legal issues (e.g., deportation)	137 (30.11)	12 (18.75)	125 (31.97)	0.03
**Age in Years, n (%)**				
18-24	61 (13.41)	18 (28.12)	43 (11.00)	<0.01
25-34	185 (40.66)	25 (39.06)	160 (40.92)	
35-44	178 (39.12)	20 (31.25)	158 (40.41)	
45 or older	31 (6.81)	1 (1.56)	30 (7.67)	
**Child Age in Years, n (%)**				
0-4	235 (51.65)	36 (56.25)	199 (50.90)	0.42
5-17	220 (48.35)	28 (43.75)	192 (49.10)	
**Highest Education Completed, n (%)**				
Less than High School	264 (58.02)	12 (18.75)	252 (64.45)	<0.01
High school/GED	127 (27.91)	28 (43.75)	99 (25.32)	
Technical school/Some College	40 (8.79)	18 (28.12)	22 (5.63)	
College Graduate/Graduate school	24 (5.27)	6 (9.38)	18 (4.60)	
**Employment Status, n (%)**				
Employed	294 (64.62)	46 (71.88)	248 (63.43)	0.19
Unemployed	161 (35.38)	18 (28.12)	143 (36.57)	
**PROMIS Physical Health Rating, n (%)**				
Good/Very Good/Excellent	287 (63.08)	35 (54.69)	252 (64.45)	0.13
Poor/Fair	168 (36.92)	29 (45.31)	139 (35.55)	

### Primary Outcomes

#### Emotional Problems

In the fully adjusted multinomial model, having any number of social needs predicted significantly higher odds of being often or always bothered by emotional problems, compared with rarely or never being bothered, (1 to 3 needs: OR = 5.89; 95% CI: 1.28, 26.92; p = 0.02; 4 or more needs: OR = 15.03; 95% CI: 3.31, 68.23; p < 0.01) (Table 2). Spanish language preference predicted lower odds of often or always being bothered by problems (OR = 0.21; 95% CI: 0.08, 0.51; p < 0.01). In the adjusted multinomial models using separate social needs and language as independent variables, all needs were significantly associated with greater odds of being often/always bothered by emotional problems compared with rarely/never being bothered (p < 0.05), except for legal issues (see Table 3 for detailed results). Spanish language preference again predicted lower odds of being often/always bothered by emotional problems, across all models (OR range 0.23–0.26; p < 0.01; results not shown in table).

**Table 2:** Multinomial Regression Model of Emotional Problems on Household Count of Social Needs and Language (N=455)

	Sometimes Bothered				Often/Always Bothered		
	**OR**	**95% CI**	**p**		**OR**	**95% CI**	**p**
**Household Count of Social Needs**							
None=ref	---	---	---		---	---	---
1-3 needs	2.12	(0.97, 4.63)	0.06		5.89	(1.28, 26.92)	0.02
4 or more needs	4.36	(1.98, 9.58)	<0.01		15.03	(3.31, 68.23)	<0.01
**Preferred Language**							
English=ref	---	---	---		---	---	---
Spanish	0.31	(0.14, 0.70)	<0.01		0.21	(0.08, 0.51)	<0.01
**Age in Years**							
18-24=ref	---	---	---		---	---	---
25-34	1.32	(0.64, 2.72)	0.43		1.42	(0.57, 3.50)	0.44
35-44	0.70	(0.33, 1.47)	0.35		1.06	(0.42, 2.68)	0.88
45 or older	0.49	(0.15, 1.54)	0.22		0.98	(0.25, 3.78)	0.98
**Child Age in Years**							
0-4=ref	---	---	---		---	---	---
5-17	1.86	(1.14, 3.06)	0.01		1.44	(0.79, 2.60)	0.22
**Highest Education Completed**							
Less than high school=ref	---	---	---		---	---	---
High school/GED	0.68	(0.40, 1.16)	0.16		0.66	(0.35, 1.26)	0.21
Technical school/Some college	0.79	(0.33, 1.89)	0.60		0.66	(0.23, 1.87)	0.44
College graduate/Graduate school	0.76	(0.27, 2.14)	0.60		0.54	(0.15, 1.94)	0.35
**Employment Status**							
Employed=ref	---	---	---		---	---	---
Unemployed	1.38	(0.85, 2.24)	0.18		1.24	(0.69, 2.23)	0.45
**PROMIS Physical Health Rating**							
Good/Very good/Excellent=ref	---	---	---		---	---	---
Poor/Fair	1.96	(1.20, 3.22)	<0.01		3.29	(1.87, 5.78)	<0.01

ref=reference group; OR=odds ratio; CI=confidence interval; p=level of significance

**Table 3remove bold from table header:** Multinomial Regression Model of Emotional Problems on Most Frequently Reported Social Needs, N=455)^a^

		Sometimes Bothered										Often/Always Bothered						
		**OR**		**95% CI**		**p**					**OR**			**95% CI**		**p**		
**Most Frequently Reported Social Needs (no=ref)**																		
Problems paying bills		2.43		(1.51, 3.89)		<0.01					2.57			(1.48, 4.45)		<0.01		
Unstable housing		1.67		(1.05, 2.66)		0.02					2.03			(1.18, 3.49)		0.01		
Habitability problems		1.93		(1.16, 3.22)		0.01					2.40			(1.34, 4.30)		<0.01		
Running out of food		2.43		(1.50, 3.93)		<0.01					2.32			(1.33, 4.07)		<0.01		
Difficulty finding a job		1.60		(0.98, 2.63)		0.06					1.90			(1.07, 3.37)		0.02		
Difficulty affording transportation		1.89		(1.15, 3.11)		0.01					2.13			(1.20, 3.79)		<0.01		
Other legal issues		1.41		(0.86, 2.29)		0.16					1.51			(0.85, 2.69)		0.15		

ref=reference group; OR=odds ratio; CI=confidence interval; p=level of significance

^a^Results reported are for separate models of the outcome on each independent variable. All models also included primary language and all covariates (not shown). Spanish language significantly predicted lower odds of being often/always bothered by emotional problems, across all above models (OR range 0.23 – 0.26; p < 0.01; not shown).

#### Perceived Stress

In the first adjusted multinomial model of perceived stress (Table 4), report of 4 or more household social needs significantly predicted greater odds for a high PSS-4 score (9 or greater), compared with having a low PSS-4 score (0 to 4) (OR = 4.97; 95% CI: 1.87, 13.91; p < 0.01). In separate adjusted multinomial models, each social need predicted greater odds of high perceived stress score compared with low (p < 0.05), except for legal issues (see Table 5 for detailed results). Preferred language was not significantly associated with perceived stress in any models (not all results shown in tables).

**Table 4:** Multinomial Regression Model of Perceived Stress on Household Count of Social Needs and Language, N=455)remove bold from table header

	PSS-4 Score 5 - 8				PSS-4 Score 9 - 20		
	**OR**	**95% CI**	**p**		**OR**	**95% CI**	**p**
**Household Count of Social Needs**							
None=ref	---	---	---		---	---	---
1-3 needs	1.15	(0.51, 2.58)	0.72		2.17	(0.81, 5.79)	0.12
4 or more needs	1.83	(0.81, 4.12)	0.14		4.97	(1.87, 13.19)	<0.01
**Preferred Language**							
English=ref	---	---	---		---	---	---
Spanish	0.78	(0.36, 1.66)	0.52		0.62	(0.29, 1.33)	0.22
**Age in Years**							
18-24=ref	---	---	---		---	---	---
25-34	0.45	(0.21, 0.96)	0.04		0.89	(0.39, 2.01)	0.78
35-44	0.44	(0.20, 0.96)	0.04		0.62	(0.26, 1.45)	0.27
45 or older	0.19	(0.05, 0.64)	<0.01		0.24	(0.06, 0.86)	0.02
**Child Age in Years**							
0-4=ref	---	---	---		---	---	---
5-17	1.14	(0.68, 1.91)	0.61		1.45	(0.87, 2.41)	0.15
**Highest Education Completed**							
Less than high school=ref	---	---	---		---	---	---
High school/GED	1.06	(0.61, 1.85)	0.81		0.74	(0.42, 1.30)	0.29
Technical school/Some college	1.86	(0.75, 4.57)	0.17		0.97	(0.37, 2.51)	0.95
College graduate/Graduate school	1.73	(0.55, 5.37)	0.34		1.30	(0.42, 3.97)	0.64
**Employment Status**							
Employed=ref	---	---	---		---	---	---
Unemployed	1.17	(0.71, 1.94)	0.53		0.88	(0.52, 1.47)	0.62
**PROMIS Physical Health Rating**							
Good/Very good/Excellent=ref	---	---	---		---	---	---
Poor/Fair	2.82	(1.69, 4.72)	<0.01		2.44	(1.46, 4.08)	<0.01

ref=reference group; OR=odds ratio; CI=confidence interval; p=level of significance

**Table 5:** Multinomial Regression Model of Perceived Stress on Most Frequently Reported Social Needs, N=455)^a^

		5-8				9 - 20		
		**OR**	**95% CI**	**p**		**OR**	**95% CI**	**p**
**Most Frequently Reported Social Needs (no=ref)**								
Problems paying bills		1.24	(0.75, 2.03)	0.38		2.13	(1.32, 3.44)	<0.01
Unstable housing		1.19	(0.73, 1.94)	0.47		1.61	(1.00, 2.60)	0.04
Habitability problems		1.32	(0.77, 2.26)	0.30		2.05	(1.23, 3.43)	<0.01
Running out of food		1.67	(1.00, 2.78)	0.04		2.75	(1.68, 4.50)	<0.01
Difficulty finding a job		1.32	(0.77, 2.26)	0.29		2.36	(1.42, 3.93)	<0.01
Difficulty affording transportation		1.43	(0.84, 2.42)	0.18		2.17	(1.31, 3.60)	<0.01
Other legal issues		1.76	(1.04, 2.95)	0.03		1.41	(0.84, 2.36)	0.19

ref=reference group; OR=odds ratio; CI=confidence interval; p=level of significance

^a^Results reported are for separate models of the outcome on each independent variable. All models also included primary language and all covariates (not shown).

## Discussion

Our study is the first to our knowledge to test associations between household social needs (individually or collectively) and symptoms of emotional problems and stress in Latinx mothers. The findings revealed significant emotional problems and stress in the sample. Over 22% of mothers in the sample reported often or always being bothered by problems of anxiety, irritability, or depression in the prior week. This is higher than national data of the same period, in which an estimated 9% of Latinx adults (not limited to mothers) reported regularly having “feelings of worry, nervousness, or anxiety” and 4% reported regularly “having feelings of depression” [46]. Over 31% of our sample had a PSS-4 score over 8; this indicates that almost one-third of our sample was experiencing high stress, based on levels ranging from 4 to 8 used in previous studies as potential thresholds for high stress [47, 48].

High count of social needs was a strong predictor of both emotional problems and perceived stress. Utility bills, food, housing, habitability, employment, transportation, and legal support were the most reported concerns in our sample, consistent with national prevalence data of the same period [49], and most of these needs also independently increased the odds for emotional problems and perceived stress. These findings are unique; though food insecurity has been extensively studied and found to be independently associated with serious psychological distress in parents with low income [49–51] and Latinx populations [52, 53], little prior evidence has demonstrated associations between other needs and emotional health in Latinx mothers. The data for this study were collected prior to the COVID-19 pandemic, which significantly widened health and economic disparities for the Latinx community [54]. If performed today, our study could yield data that reveal even greater psychological impacts of social needs on Latinx mothers. We were unable to examine causal relationships in this cross-sectional study, though there are likely bidirectional influences between economic disadvantage and emotional dysfunction [49, 55, 56]. Future work should explore the mechanisms linking these factors in the Latinx population.

The strong associations between social needs and emotional problems in our study support the need for social risk-targeted mental health care, a proposed strategy for improving health outcomes [57]. Current clinical practice guidelines independently recommend screening for depression [58] and social determinants of health [59] in adult primary care, though they do not clearly articulate which social needs should be included in these assessments (beyond income or education status). Future recommendations that link maternal mental health and material deprivation screening initiatives, not only in medical or behavioral health settings, but also in social service agencies, could strengthen identification and targeted interventions. More work is needed to understand the pathways and mechanisms underlying the associations between social needs and mental health, particularly in low-income Latinx populations, to further these efforts.

Another important finding from our analyses was that being less acculturated, as per language preference proxy, predicted lower odds of emotional problems. This association may be a function of social support, an important facet of many Latinx cultures that appears to be stronger in less acculturated individuals [17] and has been shown to lessen emotional distress in Latinx mothers [19, 60]. Future research using multidimensional measures of acculturation and cultural influence could help unpack the relationships between social needs, acculturation, and cultural considerations, including potential benefits from protective cultural factors represented by lower acculturation. This work should help to inform whether clinic-based interventions to address the mental health and social needs of Latinx mothers could be strengthened by initiatives that focus on known beneficial cultural influences such as social supports [61, 62].

We were surprised that acculturation did not similarly impact perceived stress. One explanation is that constructs assessed by the single PROMIS question and PSS-4 differ. The PSS-4 asks the participant to report on feelings of control or capacity related to life stressors [38], whereas the PROMIS question asks about the level of bother caused by emotional symptoms [63]. In our study it is therefore possible that the protective factors associated with lower acculturation are more closely related to the participant’s experience of bother related to their emotional symptoms, rather than their confidence in handling stress. This concept may be further impacted by stigma in the Latinx community that the inability to cope with mental health challenges is related to personal weakness [64], and it is possible that participants were more willing to disclose bother from emotional symptoms – a potentially more benign concept – but less so the ability to handle stress. Cultural factors may also influence the interpretation of these constructs [31], and the ethnic diversity of Latinx subgroups could contribute to variability in survey responses that limit the ability to detect significant associations with stress [64, 65].

## Limitations

Our findings should be taken in the context of various limitations. First, though our sample size of Latinx participants was relatively large, the primarily low income and education level of patients seeking care in a public hospital setting limits generalizability of results to communities with similar economic disadvantage. Second, survey bias in this study may be increased because of the potential stigma associated with both mental health [32] and economic status [66]. Third, our decision to analyze a single item from the PROMIS Global scale rather than score the full ten items may reduce its criterion validity. Fourth, use of secondary data limited our ability to test other variables likely relevant to emotional problems and stress in our study population, including factors related to parenting or immigration as confounding stressors. Secondary analysis may also be less precise in measurement. For example, acculturation was not intended as a subject of interest in the original study and therefore not directly measured, and the use of language as a proxy has limitations when compared to other more multidimensional measures [21]. Lastly, we acknowledge that the Latinx population is not monolithic, and the generalizability of our findings would have been enhanced by the ability to explore potential influences of ethnicity or geographic origin [21, 67] that were not obtained in the original RCT.

## Conclusion

Social needs put Latinx mothers at significant risk for emotional problems and stress. Latinx mothers make up a large and growing percentage of the U.S. population and labor force; the impact of their mental health outcomes on the economy and community [68], as well as on their children’s health [10], cannot be ignored. More work is needed to explore the integration of social needs and mental health assessments and services for Latinx mothers. Such efforts may also be enhanced by a deeper understanding of the cultural influences that might be leveraged to tailor interventions to address unmet needs for this group.

## Data Availability

This paper uses data from a previous RCT. After publication, deidentified participant data is available by request to researchers whose proposed use of the data has been approved. Requests can be made to holly.wing@ucsf.edu, and approval is at the discretion of the primary RCT research team, with signed data use agreement.

## Code Availability

Code for data cleaning and analysis associated with current submission are available from the corresponding author on reasonable request.
